# Association of age at menarche with metabolic syndrome and its components in rural Bangladeshi women

**DOI:** 10.1186/1743-7075-9-99

**Published:** 2012-11-09

**Authors:** Shamima Akter, Subrina Jesmin, Mazedul Islam, Sayeeda Nusrat Sultana, Osamu Okazaki, Michiaki Hiroe, Masao Moroi, Taro Mizutani

**Affiliations:** 1 Health & Disease Research Center for Rural Peoples (HDRCRP), 14/15, 1st Floor, Probal Housing Ltd., Shekertak (Adjacent to Shekertak Road 1), Mohammadpur, Dhaka 1207, Bangladesh; 2 Graduate School of Medicine, Faculty of Medicine, University of Tsukuba, Tsukuba, Ibaraki 305-8575, Japan; 3 National Center for Global Health and Medicine (NCGM), 1-21-1 Toyama, Shinjuku-ku, Tokyo, 162-8655, Japan

**Keywords:** Age at menarche, Metabolic syndrome, Women, Rural Bangladesh

## Abstract

**Background:**

Early age at menarche is associated with increased risk of metabolic syndrome in both China and the West. However, little is known about the impact of age at menarche and metabolic syndrome in South Asian women, including those from low-income country, where age at menarche is also falling. The aim of the present study was to investigate whether age at menarche is inversely associated with metabolic syndrome in Bangladeshi women, who are mostly poor and have limited access to and or poor health care facilities.

**Methods:**

This community-based cross-sectional study was performed using 1423 women aged between 15–75 years from rural Bangladesh in 2009 and 2010. Metabolic syndrome was defined according to standard NCEP-ATP III criteria. Logistic regression was used to estimate the association between age at menarche and metabolic syndrome, with adjustment of potential confounding variables, including age, education, marital status, tobacco users, use of contraceptives and number of pregnancies.

**Results:**

Early onset of menarche (<12 years) as compared to late onset (>13 years) was found to be associated with a higher prevalence of metabolic syndrome (odds ratio=1.55; 95 % confidence interval =1.05-2.30). Age at onset of menarche was also inversely associated with prevalence of high triglycerides (*P* for trend <0.01) and low high-density lipoprotein cholesterol (*P* for trend = 0.01), but positively associated with prevalence of high fasting blood glucose (*P* for trend =0.02). However, no significant association was found between age at menarche, high blood pressure and elevated waist circumference.

**Conclusion:**

Early onset of menarche might promote or trigger development of metabolic syndrome. Thus, knowledge of the history of age at onset of menarche may be critical in identifying women at risk of developing metabolic syndrome and those likely to benefit the most from early interventions.

## Background

Metabolic syndrome is a cluster of risk factors that include obesity, insulin resistance, dyslipidemia, and hypertension. Collectively, these factors increase the risk for cardiovascular disease and type 2 diabetes [1,2]. Over the past two decades, there has been a noticeable world-wide increase in the prevalence of metabolic syndrome, including more recently in developing countries, where the prevalence has been much lower [3,4]. This new development has further compounded the challenges that the weak health system in developing countries is still struggling to cope with, i.e., communicable diseases [5]. The prevalence of metabolic syndrome in women tend to be greater than in men in many developing countries [4]. In view of these gender disparities, it is critical that we identify women at risk of contracting these diseases earlier on in life for effective interventions and outcomes.

Menarche, the onset of menstruation in girls, indicates the attainment of full reproductive capacity. The age that menarche occurs varies and is dependent on the interaction between genetic and environmental factors and, in some cases, lifestyle factors [6,7]. The life style changes resulting from industrialization, decreased levels of physical activity and increased or excess intake of energy substrates has led to a rapid decrease in age at onset of menarche both in developed and more recently in developing countries [8-10]. Early onset of menarche has been shown to be associated with higher body mass index (BMI) [11-14], risk factor of cardiovascular diseases and its associated morbidity and mortality [15-17], as well as type 2 diabetes [18,19]. Therefore, in recent times, there has been increased interest in identifying whether an association exists between age at onset of menarche and metabolic syndrome, in order to better prevent and manage the myriad chronic non-communicable diseases that occur in women.

To date, few studies have reported the association between age at onset of menarche and metabolic syndrome [16,20,21] and the bulk of the data from these studies were mainly obtained from the West [16,21], with very limited data from Asia [20]. Specifically, to our knowledge, no such study has been conducted so far on this issue in South Asian population or in low-income countries, including Bangladeshi women. The characteristics and conditions of South Asian women are substantially different from Western or Far East Asian populations. For instance, South Asian women tend to have a higher prevalence of abdominal obesity but lower BMI compared to Western and other Asian women [4]. Further, there are socio-economic disparities between the different regions of the globe. The current mean age at menarche is 12.8 years in Bangladesh, which was considerably lower than the previous estimate [22]. Bangladesh is a low-income country with a GDP per capita income of 588 USD, an average life expectancy at birth of 67 years, and an adult literacy rate of about 55% [23]. Besides, in Bangladesh more than one-third of the population lives below the poverty lines and are unable to even meet the barest of their basic needs [24], and women disproportionately constitute the bulk of the poor community. Thus, the goal of the present study was to clarify whether age at onset of menarche plays any etiological role in the development of metabolic syndrome in a low-income country by investigating existing of an association between age at onset of menarche and metabolic syndrome and its components. We hypothesized that age at onset of menarche is inversely associated with metabolic syndrome in rural Bangladeshi women.

## Methods

### Study procedure

The present study is a community-based cross-sectional study performed using women from rural Bangladesh between 2009 and 2010. A total of 1535 females aged ≥15 years were selected using the stratified multistage random sampling. This sample size (1535) was sufficient to test all our formulated research hypotheses at the 5% level of significance, with a power of 80% (β=0.20). We used the World Health Organization’s (WHO) STEPS approach (modified), which entails a stepwise collection of the risk factor data, based on standardized questionnaires covering demographic characteristics, somatic illnesses, somatic and mental symptoms, medications, life style, and health-related behavior (step 1), basic physical measures (step 2) and basic biochemical investigations, such as blood glucose and cholesterol (step 3). The women were recruited from 4 village communities located in Gabindagonj Upazilla (sub district) of Gaibandha district. The respondents were selected randomly after selecting the division, district, Upazila and villages, and were recruited through local announcements at community level and by house-to-house visits. The details of the study area have been described before in our previous study [25]. Gobindogonj Upazilla has a population of 514,696, with average household size of about 3.88, and the main occupation is agriculture, which is similar to other rural parts of Bangladesh [26]. The literacy rate of our target community is 45.8% and 39.5% for males and females, respectively, which is a bit lower than the average or overall rural literacy rate (54.39% and 46.19% for male and female, respectively) in the country [26]. The data from participants were obtained through interviews and clinical examinations at mobile examination centers, where blood samples were also collected. The study was approved by the Ethical Committee of the Health and Disease Research Center of Rural Peoples (HDRCRP), Dhaka, Bangladesh, and conforms to the principles outlined in the Helsinki Declaration. Also all participating subjects gave their written informed consent before they were included in the study.

### Study subjects

Out of 1535 women, the following subjects were excluded: those lacking information on triglyceride, high-density lipoprotein (HDL) cholesterol, and fasting blood glucose; missing information of age at onset of menarche, or missing information for any other covariates used in the main analysis. Participants with menarche later than 16 years were also excluded, because this is likely to be due to pathological status or recall error. Finally, a total of 1423 subjects remained in this study.

### Anthropometric and other variables

Anthropometric measurements on individuals wearing light clothing and without shoes were conducted by well-trained examiners, as described here: height was measured to the nearest 0.1 cm using the portable stadiometer; weight was then measured in an upright position, to the nearest 0.1 kg, using a calibrated balance beam scale; BMI was calculated as body weight (kg) divided by the square of the body height (m^2^); and waist circumference measurements were taken at the end of normal expiration, to the nearest 0.1 cm, by measuring from the narrowest point between the lower borders of the rib cage and the iliac crest. Blood pressure was measured twice in the right arm in a sitting position using the standard mercury manometer and cuff, to the nearest 2 mmHg, with the initial reading taken at least 5 minutes after the subject was made comfortable, and again after an interval of 15 minutes. The average systolic and diastolic blood pressures were then estimated. Tobacco use, education, marital status, use of contraceptives, and experience of prior pregnancies were self-reported. Ever tobacco users were defined as those who are current tobacco users (smoked/chewed tobacco) and who had not smoked/chewed tobacco in the past 30 days preceding the survey, but have prior experience.

### Biochemical analysis

Blood for biochemical analysis was obtained from the participants after a 10–12 hour overnight fast. The blood sample was collected using the standard blood sample collection procedure. Immediately after collection of blood and labeling of the blood vials, the samples were transported to the National Centre for Global Health and Medicine (NCGM), Japan, for biochemical assessment. For analysis, the serum was immediately separated from the blood by centrifugation for evaluating plasma concentration of lipids. Triglycerides levels were measured by lipoprotein lipase method (Wako Chemicals, Tokyo, Japan), HDL cholesterol was measured with the Determiner-L kit (Kyowa Co Ltd, Tokyo, Japan). Fasting plasma glucose levels were measured with the Hexokinase G-6-PDH kit (Wako Pure Chemical Industries Ltd, Osaka, Japan).

### Assessment of age at menarche

Age at onset of menarche was defined as age at the first menstrual bleeding, assessed in full years. This information was obtained by a personal interview at the time of survey. The question was open ended “at what age did you have your first menstrual period (menarche)?” For the present study, analysis of age at onset of menarche was categorized into three categories (<12 years, 12–13 years, and >13 years). The reliability of the questionnaire on age at onset of menarche was confirmed in our follow up study. The intra-class correlation coefficient was 0.89 for continuous variables of age at onset of menarche.

### Definition of metabolic syndrome and risk factors

Metabolic syndrome and metabolic risk factors were defined according to standard criteria of the National Cholesterol Education Program’s Adults Treatment Panel III (NCEP-ATP III) [27]. Three or more of the following components were defined as having metabolic syndrome, namely: a) abdominal obesity, as measured by a waist circumference of ≥88 cm ; b) high fasting blood glucose (≥110 mg/dL or ≥6.1 mmol/L) or patients diagnosed with diabetes; c) high triglycerides (≥150 mg/dL or ≥1.7 mmol/L); d) low HDL cholesterol (<50 mg/dL or <1.29 mmol/L); e) high blood pressure (≥130/≥85mmHg) or subjects diagnosed with hypertension. Also, participants who at the time of the study reported to be on anti-hypertensive or anti-diabetic medications (insulin or oral agents) were considered as having high blood pressure or high fasting blood glucose, respectively.

### Statistical analysis

The characteristics of the participants of the study were presented according to increasing categories of age at onset of menarche and trend associations were assessed using linear regression analysis for continuous variables or Mantel-Haenszel chi-square test for categorical variables, with ordinal numbers 1–3 assigned to increasing categories of age at menarche.

To evaluate the magnitude of the association between age at onset of menarche and metabolic syndrome and its components (obesity, high triglycerides, low HDL cholesterol, high blood pressure, and high fasting blood glucose), we estimated adjusted odds ratio (OR) and 95% confidence interval with multivariable logistic regression models. Two models were used considering highest age at onset of menarche category as reference category. The first models were adjusted for age (year, continuous), and education (illiterate, have formal education). The second models were further adjusted for marital status (currently married or others), tobacco users (ever or never), use of contraceptives (ever or never), and number of pregnancies (continuous). Trend association was assessed by assigning ordinal numbers 1–3 assigned to increasing categories of age at menarche. Two-sided *P* values <0.05 were regarded as statistically significant. All analyses were performed using statistical software STATA version 12.0 (Lakeway Drive, College Station, Texas USA).

## Results

Table 1 shows the characteristics of the study subjects, based on the categories of age at onset of menarche. Age at onset of menarche was positively associated with levels of fasting blood glucose and HDL cholesterol but was inversely associated with levels of triglyceride. Subjects with higher age at menarche were more likely to be currently married and have no formal education but less likely to be tobacco users. Prevalence of metabolic syndrome across categories of age at onset of menarche was also presented (Table 1). As age at onset of menarche increased, prevalence of metabolic syndrome was found to decrease, although it was not found to be statistically significant (*P* for trend = 0.16).

**Table 1:** Characteristics of study population by age at menarche

**Characteristic**	**Age at menarche (years)**			
	**<12**	**12-13**	**>13**	**Trend P**^**b**^
n	488	583	352	
Current age (years)	40.91±11.68^a^	42.12 ± 12.92	42.72 ± 13.57	0.04
BMI (kg/m^2^)	21.43 ± 4.94	21.96 ± 4.01	22.26 ± 4.05	0.08
Number of pregnancies	3.50 ± 2.13	3.44 ± 2.08	3.36 ± 2.03	0.35
Currently married (%)	80.94	88.16	94.32	<0.01
Ever use of contraceptives (yes, %)	19.67	17.50	19.03	0.74
Use of tobacco products (ever, %)	31.56	19.04	7.10	<0.01
Education (illiterate, %)	52.05	54.37	59.66	0.03
Waist circumference (cm)	77.20 ± 9.32	76.96 ± 8.86	76.38 ± 7.90	0.19
Fasting blood glucose (mmol/L)	5.81 ± 2.12	6.29 ± 2.99	6.78 ± 4.65	<0.01
Triglyceride (mg/dL)	131.5 ± 102.8	130.5 ± 105.4	127.9 ± 119.2	0.03
HDL cholesterol (mg/dL)	36.54 ± 18.7	40.89 ± 31.05	48.71 ± 42.40	<0.01
Systolic blood pressure (mmHg)	115.46 ± 20.26	116.73 ± 21.74	116.70 ± 19.46	0.28
Diastolic blood pressure (mmHg)	74.53 ± 10.00	75.74 ± 10.34	75.29 ± 10.53	0.23
Metabolic syndrome (%)	26.14	25.73	22.13	0.16

Abbreviation: BMI, body mass index; HDL, high-density lipoprotein.

^a^Values are mean±SD, all such values.

Table 2 shows odds ratio of metabolic syndrome and its components, according to increasing categories of age at onset of menarche. Age at onset of menarche was inversely associated with prevalence of metabolic syndrome in age and education adjusted model (*P* for trend = 0.02). Additional adjustment for other covariates, including marital status, tobacco and contraceptive usage, and number of pregnancies, were found to somewhat attenuates the association but still found to be statistically significant (*P* for trend = 0.03). Subjects with the lowest age at onset of menarche had 1.55 times higher odds of having metabolic syndrome than those with late onset of menarche. Age at onset of menarche was also inversely associated with prevalence of high triglyceride (*P* for trend <0.01) and low HDL cholesterol (*P* for trend = 0.01) but was found to be positively associated with high fasting blood glucose (*P* for trend = 0.02). High blood pressure and elevated waist circumference was not significantly associated with age at onset of menarche (*P* for trend = 0.83 and 0.23, respectively).

**Table 2:** Association of age at menarche with metabolic syndrome and its components

	**Age at menarche (years)**			
	**<12**	**12-13**	**>13**	**Trend P**^**a**^
Metabolic syndrome^b^				
Model 1	1.60 (1.09−2.35)	1.38 (0.97−1.97)	1.00 (reference)	0.02
Model 2	1.55 (1.05−2.30)	1.36 (0.95−1.94)	1.00 (reference)	0.03
Central obesity				
Model 1	1.67 (0.92−3.05)	1.82 (1.02−3.27)	1.00 (reference)	0.11
Model 2	1.53 (0.82−2.85)	1.73 (0.96−3.11)	1.00 (reference)	0.23
High fasting blood glucose				
Model 1	0.59 (0.42−0.83)	0.73 (0.54−0.99)	1.00 (reference)	<0.01
Model 2	0.65 (0.46−0.93)	0.76 (0.56−1.04)	1.00 (reference)	0.02
High triglyceride				
Model 1	2.27 (1.59−3.25)	1.82 (1.31−2.53)	1.00 (reference)	<0.01
Model 2	1.97 (1.36−2.84)	1.70 (1.22−2.35)	1.00 (reference)	<0.01
Low HDL cholesterol				
Model 1	1.99 (1.32−2.98)	1.41 (1.00−2.00)	1.00 (reference)	<0.01
Model 2	1.71 (1.12−2.60)	1.30 (0.91−1.84)	1.00 (reference)	0.01
High blood pressure				
Model 1	0.94 (0.66−1.35)	1.01 (0.73−1.43)	1.00 (reference)	0.71
Model 2	1.05 (0.72−1.52)	1.08 (0.77−1.53)	1.00 (reference)	0.83

Model 1 adjusted for age (year, continuous), education (illiterate, have formal education).

Model 2 adjusted for age (year, continuous), education (illiterate, have formal education), marital status (currently married or others), use of tobacco products (ever or never), ever use of contraceptives (yes or no), and number of pregnancies (continuous).

^a^Based on multiple logistic regression analysis, with ordinal numbers 1–3 assigned to increasing categories of age at menarche.

^b^Metabolic syndrome is defined as presence of at least 3 of the following criteria.

Obesity component (Waist circumference ≥88 cm), high fasting blood glucose (≥110 mg/dL), high triglycerides (≥150 mg/dL), low-high-density lipoprotein (HDL) cholesterol (<50 mg/dL), high blood pressure; systolic blood pressure (SBP ≥ 130mm Hg) or diastolic blood pressure (DBP ≥85 mm Hg).

## Discussion

In the present cross-sectional study, we use rural Bangladeshi women to examine the relationship between age at onset of menarche and metabolic syndrome. We found that age at onset of menarche was inversely associated with metabolic syndrome in these women after controlling for potential confounding variables. Age at onset of menarche was also inversely associated with some components of metabolic syndrome, including high triglyceride, and low HDL cholesterol, but was found to be positively associated with high fasting blood glucose. To our knowledge this is the first study to reveal an association between age at onset of menarche and metabolic syndrome in South Asian women and in a low-income country.

The inverse association between age at onset of menarche and metabolic syndrome are consistent with most of the previous studies from both the Western [16,21] and Far Eastern Asian populations [20]. The present findings are also in agreement with two previous studies of same line conducted in the US [28] and China [29]. Therefore, the present data reconfirms these earlier findings and demonstrate their prevalence in Bangladeshi women, who are mostly poor, have lower BMI but higher waist circumference and have lower mean age at menarche compared with their Western and Far Eastern Asian counterparts [4,22], suggesting that early onset of menarche may increase prevalence of metabolic syndrome, irrespective of race and ethnicity.

Age at onset of menarche was inversely associated with high levels of plasma triglyceride, and low HDL cholesterol components in our study. The significant inverse association between age at onset of menarche and high triglycerides in our study are concordant with those of the previous studies [20,21,29], conducted so far. Data are sparse and conflicting as to whether early onset of menarche is inversely associated with increasing prevalence of low HDL cholesterol. The inverse association between age at onset of menarche and low HDL cholesterol revealed in our study is consistent with a previous Chinese study [29]. However, the majority of previous studies found no significant association between age at onset of menarche and prevalence of low HDL cholesterol [20,21]. Although currently there is no clear evidence that early onset of menarche increases the prevalence of low HDL cholesterol, based on the present findings, we could expect to see significant benefits in late onset of menarche in as far as metabolic syndrome prevalence is concerned, as well as lower prevalence of HDL cholesterol. Such trends and associations also provides us with some important insights that may play a crucial role in the development of metabolic syndrome preventive interventions in these rural communities where prevalence of low HDL cholesterol is significantly high [30].

Unlike the expected association between age at onset of menarche and other metabolic components, age at onset of menarche was not inversely associated rather positively associated with fasting blood glucose. These results have not been reported previously so far by earlier studies. Contrarily to the findings of the present studies, most of the previous studies either found no significant association [16,29,31] or inverse association between age at onset of menarche and high fasting blood glucose [21,28,32]. In addition, in a previous Chinese study, a threshold effect of early menarche (<12.5 years) on elevated fasting blood glucose was found [20]. The results of our current study contract these earlier findings. At this time, there is no apparent reason to account for this association. However, it is possible that there may be some other factors responsible for this association other than age at onset of menarche alone, which may include various aspects of lifestyle of the study subjects.

The observed association between late onset of menarche and high fasting blood glucose could be attributed, in part, to the current unhealthy lifestyle and low quality diet in women with late onset of menarche. Alternatively, these present findings may reflect differences between the age groups and circumstances surrounding their generations since the subjects with late menarche were relatively older in our study population. The relatively late onset of menarche among older women are likely linked to various malnutrition factors caused either by inflammation or famine, caused by flooding or following Bangladesh’s war for independence in 1971 [33]. However, after 1976, the economy began to steadily advance. Based on stratified analysis, according to the mean current age of women (41.48 years), a clear positive association between age at onset of menarche and high fasting blood glucose was found only among older women (data not shown). However, no significant association was found among the younger women (data not shown). Therefore, collectively, these trends suggest that the lifestyle and life circumstances of the older women may be responsible for the positive association between age at onset of menarche and high fasting blood glucose, which are different from the experiences of the younger women.

The mechanism underlying the positive association between age at onset of menarche and metabolic syndrome for now remains unclear. It has been suggested that childhood obesity may influence the age at onset of sexual maturation and hence the age at the onset of menarche [34,35]. In addition, it was found that obesity in childhood is associated with metabolic syndrome in adolescence and adulthood [16,36]. Therefore, it is possible that the observed association between early onset of menarche and metabolic syndrome may be explained by the link between childhood obesity and metabolic syndrome. Lastly, differences in the pattern and levels of sex hormone differences over the lifespan of the women may account for the observed association between early menarche and metabolic syndrome. This speculation warrants a prospective study for clarity.

The major strengths of the present study include the fact that it is based on: a) a community-wide survey drawn from the general population, b) the anthropometric data are derived from actual measurements rather than self-reported, c) and are adjusted for potential confounding variables. However, despite these strengths, the present study has some limitations that are worth mentioning: First, since the ages at onset of menarche were self-reported, there could have been some error in reporting. However, the extent of such an error should have been minimal since onset of menarche is a discrete physiological event and a key milestone in the lives of girls, thus continues to be memorable event even in adulthood. This conclusion is consistent with data from previous studies that showed that the actual age at onset of menarche and recall of age at menarche was not found to differ even after 33 years later [11,37]. The second limitation is that it was not possible to adjust for pubertal BMI due to a lack of information of the self-reported BMI at the onset of menarche or menstruation. This is because in a low-income country like Bangladesh, rural women and girls are not always aware about their BMI, so it is difficult to assess the accuracy of self-reported pubertal BMI. Thirdly, although we adjusted for important confounders, the possibility of residual confounding cannot be completely ruled out. Finally, since our study is a cross-sectional it could have selection bias during case recruitment because only rural women from lower socio-economic class were used. Thus, these results cannot be generalized to the whole community of Bangladeshi women nation-wide. However, because there are minimal differences in rural communities, the present results may relevant to the larger nation-wide community of Bangladeshi women in rural areas.

In conclusion, the present study shows that early onset of menarche is associated with metabolic syndrome in rural Bangladeshi women. Knowledge of the history of age at onset of menarche may be essential and help in identifying women at risk or those likely to have metabolic syndrome later in life. Early identification of women at risk may help to prevent metabolic syndrome, which is also the precursor of type 2 diabetes and cardiovascular diseases.

## Abbreviations

BMI: Body mass index; HDL cholesterol: High-density lipoprotein cholesterol.

## Competing interests

The authors declare that they have no competing interests**.**

## Authors’ contributions

The authors responsibilities are as follows- SA: conducted data analysis, drafted the manuscript and had primary responsibility for final content; SJ: conducted data collection, arranged financial support, extensively reviewed and edited the manuscript; and all authors: were involved in interpretation of results and revision of the manuscript and approved the final version of the manuscript.
